# New Reference Genes for qRT-PCR Analysis as a Potential Target for Identification of *Trichophyton verrucosum* in Different Culture Conditions

**DOI:** 10.3390/pathogens10111361

**Published:** 2021-10-21

**Authors:** Sebastian Gnat, Dominik Łagowski, Aneta Nowakiewicz, Aleksandra Trościańczyk, Mariusz Dyląg

**Affiliations:** 1 Department of Veterinary Microbiology, Faculty of Veterinary Medicine, University of Life Sciences, Akademicka 12, 20-033 Lublin, Poland; aneta.nowakiewicz@up.lublin.pl (A.N.); aleksandra.troscianczyk@up.lublin.pl (A.T.); 2 Department of Mycology and Genetics, Faculty of Biological Sciences, University of Wroclaw, 51-148 Wroclaw, Poland; mariusz.dylag@uwr.edu.pl

**Keywords:** qRT-PCR technique, housekeeping genes, *Trichophyton verrucosum*, gene expression

## Abstract

Dermatophytes are a group of filamentous fungi infecting skin, hair, and nails that raise great diagnostic difficulties. qRT-PCR is a reliable technique for quantifying gene expression with increasingly frequent use in mycological diagnostics. Knowledge of genes and molecular markers with potential to be used in the identification of dermatophytes is of great importance for the development of this branch of diagnostics. In this article, the suitability of six candidate reference genes (*TUBB*, *ACTB*, *ADPRF*, *RPL2*, *SDHA*, and *EEF1A1*) was investigated for gene expression analysis in the dermatophyte *Trichophyton verrucosum*, which was cultured in various mycological media that are commonly used in a diagnostic laboratory, i.e., Sabouraud, potato dextrose, and keratin-supplemented MM-Cove. The different culture conditions are extremely important factors for the growth and physiology of dermatophytes. Gene expression stability was evaluated using geNorm, NormFinder, BestKeeper, and RefFinder algorithms. Regarding the stability of expression, *SDHA* was the most stable housekeeping gene; hence, this gene is recommended for future qRT-PCR studies on *T. verrucosum* strains. These results allow us to conclude that the *SDHA* gene can be an additional good candidate as an identification target in the qRT-PCR technique.

## 1. Introduction

Dermatophytes are a group of pathogenic fungi that can invade keratinized structures, leading to infection of skin, hair, and nails [1]. Currently, dermatophytes encompass more than 50 species belonging to the genera *Trichophyton*, *Microsporum*, *Epidermophyton*, *Arthroderma*, *Nannizzia*, *Lophophyton*, and *Paraphyton* [1,2]. The optimal conditions for dermatophyte growth are a warm and humid climate; hence, they are frequently noted in tropical and subtropical regions [3,4,5]. Therefore, dermatomycoses are more prevalent in many African and Mediterranean countries, as well as in other countries with warm climates worldwide, than in countries with colder climates [5,6]. However, an increasing number of dermatomycoses, including cases that are difficult to treat, are being documented in the scientific literature due to the renaissance of these diseases in developed countries [7,8,9,10]. In recent years, one such dermatophyte species with an increasing prevalence of superficial infections in humans is *Trichophyton verrucosum* [9,11,12,13]. In Europe, this increase is thought to correlate with the substantial number of cattle-rearing farms. In turn, traditional outdoor rearing with freely wandering and gathering animals may explain the acquisition and transmission of this dermatophyte species [11,12,14]. Moreover, *T. verrucosum* dermatomycosis in humans can be regarded as an occupational disease [11].

The phenomenon of the increasing number of dermatomycoses can be partially connected with difficulties in diagnosis and proper determination of the etiological factors of infection [10,13]. Currently, several methods of detecting dermatophytes are available [15,16,17,18]. One of the methods in clinical use is based on direct microscopic examination of dermatological specimens obtained from clinical lesions in dimethyl sulfoxide (DMSO) with 10% KOH, which is a rapid diagnostic tool [11,13]. However, because of its low contrast, inexperienced laboratory technicians may overlook positive diagnoses [15]. In vitro culture methods combined with microscopic examinations are rather specific diagnostic techniques; however, they are time consuming and may take up to 8 weeks to yield results [19]. Furthermore, there is a noticeable trend of developing methods based on the use of PCR as a reliable tool, which provides significantly improved efficiency in comparison with conventional mycological techniques [15,17,20,21]. The benefits of PCR methods employed for routine analysis of dermatological samples have to be balanced with the relative importance of acquisition of a result in a short amount of time, the cost of performing the analysis, the necessary equipment, and especially the time spent conducting laboratory manipulations [17,19,22,23].

The qRT-PCR techniques are progressively and frequently used for mycological diagnostic purposes [24,25]. However, they largely depend on the stability of internal reference genes used for identification of dermatophytes [26]. Therefore, sequences with the expression stability of candidate identification genes were designed for only a few molecular markers [17]. In the methods developed so far, primers specific for detection of only one dermatophyte species or for several different dermatophytes, i.e., pan-dermatophyte primers, are used [15,17]. The ITS and 28S rDNA sequences and genes encoding topoisomerase II and chitin synthase are usually used as targets [15]. The choice of other proper reference sequences that are useful in dermatophyte identification remains one of the current challenges in increasing the credibility of qRT-PCR techniques in routine diagnostics.

The aim of this study was to assess the transcription level of six housekeeping genes, which were taken into consideration to be used as identification marker sequences. The *TUBB* (encoding β-tubulin), *ACTB* (encoding β-actin), *SDHA* (encoding succinate dehydrogenase complex flavoprotein subunit A), *ADPRF* (encoding ADP ribosylation factor), *RPL2* (encoding ribosomal protein L2), and *EEF1A1* (encoding translation elongation factor-1α) genes were the subject of our interest. The suitability of the selected molecular markers in the molecular identification of *T. verrucosum* was evaluated. For comparison, selected clinical and reference strains of *T. verrucosum* were grown on the most commonly used mycological medium, i.e., Sabouraud glucose medium or potato dextrose medium, and under keratin-rich conditions typical for the natural host.

## 2. Materials and Methods

### 2.1. Dermatophyte Strains and Growth Conditions

In this study, 14 *T. verrucosum* clinical isolates were obtained from symptomatic infections in cattle (GenBank accession numbers of ITS sequences: MG251673, MG251681, MG251677, MG251671, MG251669), sheep (MG251685, MG251690, MG251691), llamas (MK369717, MK369716), and humans (MG251681, MG251688, MG251693, MK369715); one reference strain, *T. verrucosum* CBS365.53, was also used. All strains were identified with conventional phenotypic and molecular methods and have been described in detail in earlier publications [11,13]. Inoculum suspensions of each strain were prepared from 21-day-old cultures grown at 37 °C in potato dextrose medium (PDA, Oxoid, Wesel, Germany). Colonies of the tested strains were covered with sterile 0.85% NaCl with 0.025% Tween 80 added to prevent aggregation of conidia, and the suspension was prepared gently by scraping the colony surface with a sterile swab wetted in saline. Finally, the mixture of conidia and hyphae was transferred to a new tube, shaken for 20 s, and kept for sedimentation of heavy hyphal fragments for 15–20 min. The upper homogeneous suspensions were collected. Inoculum suspensions were adjusted spectrophotometrically to optical densities [OD] of approximately 10^7^ cells/mL at 550 nm. Two independent growth systems were used. In the first, the cultures were grown in a standard liquid Sabouraud glucose medium (BioMaxima, Lublin, Poland) and potato dextrose broth (Oxoid, Wesel, Germany). The second system was based on the use of liquid minimal medium (MM-Cove) [27] supplemented with keratin from cow’s hair. The keratin was obtained via the method described previously [28]. Briefly, cattle hairs were cut into ca. 1-mm fragments and defatted for 30 min at room temperature with a chloroform and methanol mixture (1:1 v:v). The material was washed in water with grey soap overnight at 42 °C, followed by a few rinses with distilled water, filtration through a sterile nitrocellulose filter, and drying at room temperature. The prepared keratin-rich substrate (as described above) was added separately and directly to a liquid minimal medium at a final concentration of 1%. The conidia were incubated separately in both a minimal and a Sabouraud liquid medium for 48 and 72 h at 37 °C with agitation.

### 2.2. RNA Extraction and cDNA Synthesis

Total RNA was extracted from *T. verrucosum* cells of clinical and reference strains using an RNeasy Plant Mini Kit (Qiagen, Hilden, Germany). The quantity and purity of the RNA was preliminary assessed spectrophotometrically on the basis of A260/A280 and A260/A230 ratios measured by NanoDrop 2000 (Thermo Fisher Scientific, Waltham, Massachusetts, USA). Then, the RNA evaluation was performed visually during electrophoresis in a 1% denaturing agarose gel. The RNA (2 μg) was reverse-transcribed into cDNA to a final volume of 40 μL using RevertAid Transcriptase (Thermo Fisher Scientific, Waltham, MA, USA). Commercial kits were used in accordance with the manufacturer’s instructions.

### 2.3. qRT-PCR Analysis

The reference genes (*TUBB*, *ACTB*, *ADPRF*, *RPL2*, *SDHA*, and *EEF1A1*) and primers were selected and designed based on the sequences obtained from the GenBank database and previous literature reports (Table 1). In addition, the Primer3 Plus software was used to design primers (http://www.bioinformatics.nl/cgi-bin/primer3plus/primer3plus.cgi, assessed on 12 March 2021). Each primer pair underwent experimental evaluation and was accepted if all of the following conditions were fulfilled: (1) the product PCR was specific, (2) PCR using genomic DNA as a template gave no product, and (3) the efficiency of RT-PCR was between 90 and 110%. qRT-PCR was performed in the RotorGene Q System (Qiagen, Hilden, Germany). The reaction mixture consisted of 10 μL of SsoAdvanced Universal SYBR^®®^ Green Supermix (2x concentrated, BioRad, Hercules, CA, USA), 1 μL of each primer (500 nM), 5 μL of diluted cDNA (1:40), and 4 μL of nuclease-free water. Amplification reactions were performed accordingly: Initial denaturation (96 °C for 60 s) followed by 40 cycles comprising the following steps: denaturation at 95 °C for 30 s, annealing at 61 °C for 30 s, and elongation at 72 °C for 20 s. A dissociation step cycle (72 °C for 15 s, and then 0.5 °C for 15 s until 95 °C) was added for melting curve analysis. All qRT-PCR experiments were performed in triplicate. The amplification efficiency (E) and correlation coefficient (R2) were calculated using the Rotor-Gene Q Series Software Version 2.3.1. (Qiagen, Hilden, Germany) using the standard curve method with 4-fold serial dilutions.

### 2.4. Data Analysis

The expression stability of the analyzed genes was evaluated in experimental conditions in four algorithms: geNorm module, NormFinder, BestKeeper, and RefFinder. The principles for comparison of the qRT-PCR parameters were as follows: (1) The geNorm module algorithm to compute the expression stability values for all genes uses Ct values. The genes were ranked according to M-value, i.e., the expression stability specified as the average pairwise variation of a particular gene with all other tested genes. The gene with the lower M-value is more stable expressed, while those with the higher M-value have less stable expression [29]. (2) The NormFinder is Visual Basic application software in which intra- and inter-group variations are taken into account and a stability value (SV) is calculated. Genes with lower SV-values present low inter- and intra-group variations, i.e., their stability is considered higher [30]. (3) The BestKeeper algorithm assess stability by calculation of standard deviation SD (±Ct) and correlation coefficients of variance CV (% Ct) for genes in all growth conditions. All stably expressed genes are combined into an r-index (coefficient of correlation) using the geometric mean of the Ct value of each gene [31]. (4) The RefFinder tool (http://leonxie.esy.es/RefFinder/ assessed on 12 March 2021) was used to combine the results and rank the genes according to the principle that the lowest rank indicates as the most stably expressed gene.

The validation of the analyzed genes was conducted using a comparison of the expression profile of the *GAPDH* gene (encoding glyceraldehyde 3-phosphate dehydrogenase) and one of the most stable genes from the six tested and selected by GeNorm, NormFinder, BestKeeper, and RefFinder in clinical isolates cultivated in keratin-supplemented MM-Cove minimal medium, Sabouraud medium, and potato dextrose agar. Three different candidate reference gene variants were tested: variant I—two least stable reference genes; variant II—three most stable reference genes; variant III—all candidate reference genes. The so-called relative expression was calculated using the 2^−ΔΔCt^ method [32].

All results were subjected to statistical analysis in R program ver. 4.0.3 (The R Foundation for Statistical Computing; Vienna, Austria). The statistical significance between the expression and conditions was analyzed with one-way ANOVA.

## 3. Results

The cDNA was obtained using total RNA isolated in three independent repetitions from cells of *T. verrucosum* strains incubated in Sabouraud, potato dextrose, and MM-Cove medium. The genes exhibited Ct values ranging from 15.0 to 19.8 (Table 1). 

**Figure 1:**
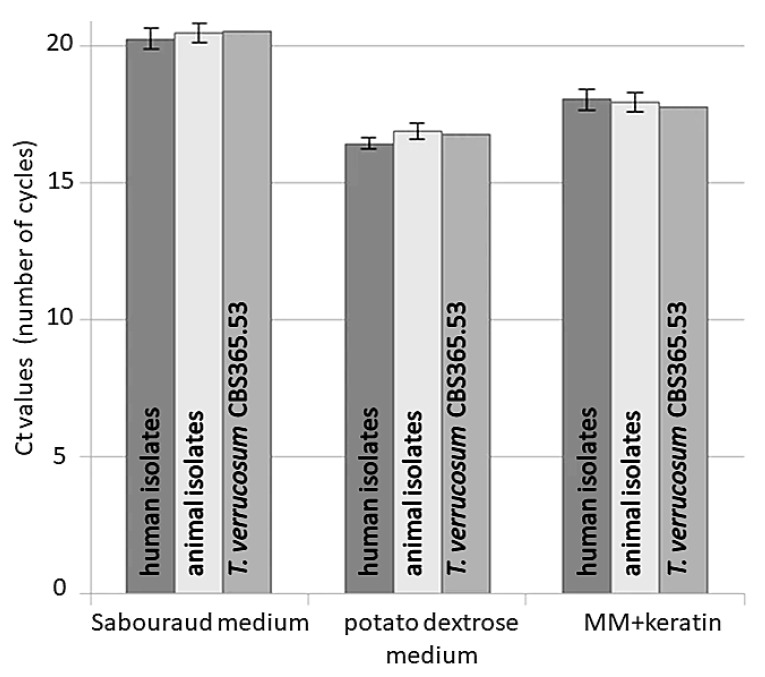
Stability of *TUBB*, *ACTB*, *ADPRF*, *RPL2*, *SDHA*, and *EEF1A1* reference genes’ expression, evaluated in three groups of *Trichophyton verrucosum* strains cultured on Sabouraud medium, potato dextrose medium, and MM-Cove medium supplemented with keratin. Levels of gene expression are shown as geometric mean of Ct values and are not significantly different across analyzed culture conditions. Error bars indicate standard deviation.

Moreover, slight variation in the Ct values was observed under the experimental conditions in various microbiological media between isolates obtained from humans, animals, and a reference strain (Figure 1). 

**Figure 2:**
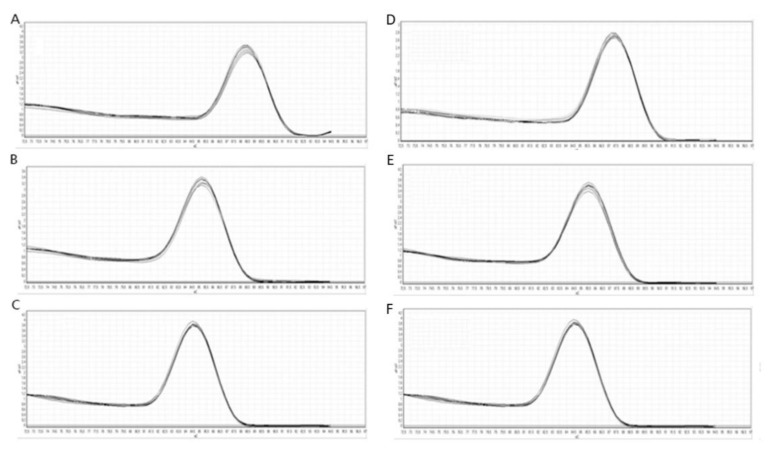
Melting curves of *Trichophyton verrucosum* genes. Gene designations: (**A**): *TUBB*, (**B**): *ACTB*, (**C**): *EEF1A1*, (**D**): *RPL2*, (**E**): *SDHA*, (**F**): *ADPRF*.

However, these differences were not statistically significant. The calculated efficiencies for the *TUBB*, *ACTB*, *ADPRF*, *RPL2*, *SDHA*, and *EEF1A1* genes were between 96% and 100%. The efficiency curves for these genes were found to have linear correlation coefficients (R2) in the range of 0.995–0.997. The melt peak analysis demonstrated a single homogenous peak for all primer sets (Figure 2). 

The GeNorm module ranks the analyzed genes based on stability value (M-value). The most stable expression profile was revealed for the *SDHA* gene, which presented a mean of the M-value equal to 0.455. On the other hand, *EEF1A1* was the least stable gene (mean of the M-value = 0.746) (Table 2). The stability analysis carried out with the geNorm module showed that all analyzed genes yielded more stable expression profiles when keratin was the sole carbon and nitrogen source during *T. verrucosum* incubation, although without tangible statistical significance of differences in gene expression. Similarly, the NormFinder analysis showed that the *EEF1A1* gene had the lowest stability value (mean of SV = 0.36). It also demonstrated that, when the samples were subjected to different culture conditions, statistically significant higher expression stability was determined in the keratin-supplemented MM-Cove medium. Moreover, the NormFinder showed that *SDHA* was the most stable gene during *T. verrucosum* cultivation in both Sabouraud and MM-Cove medium. The Excel-based BestKeeper algorithm using pairwise correlation and regression analysis assessed the inter-gene relations and revealed that all three genes exhibited an optimal standard deviation value [0.5 < SD[± Ct] ≤ 1.00] (Table 2). In addition, the lowest variation was observed for the TUBB gene (CV = 8.18). Moreover, the *TUBB* gene had the lowest correlation r-index (r = 0.78) compared to the other genes. Finally, RefFinder, a web-based tool that integrates geNorm, NormFinder, and BestKeeper, showed that the *SDHA* gene was a statistically significant top stable gene in all experimental conditions.

Furthermore, in this study, the *TUBB*, *ACTB*, *ADPRF*, *RPL2*, *SDHA*, and *EEF1A1* gene expression profiles in the case of clinical isolates of *T. verrucosum* (obtained from various hosts) were compared with those obtained for reference strain *T. verrucosum* CBS365.53, which was used for evaluation of the growth conditions. Slight variations in the Ct values were observed in the expression of all analyzed genes, although it was not significantly different in the tested conditions (Figure 2). These results confirmed the expression stability of the tested genes in strains associated with different hosts, suggesting that these genes are sufficient for effective normalization of qRT-PCR analysis.

**Figure 3:**
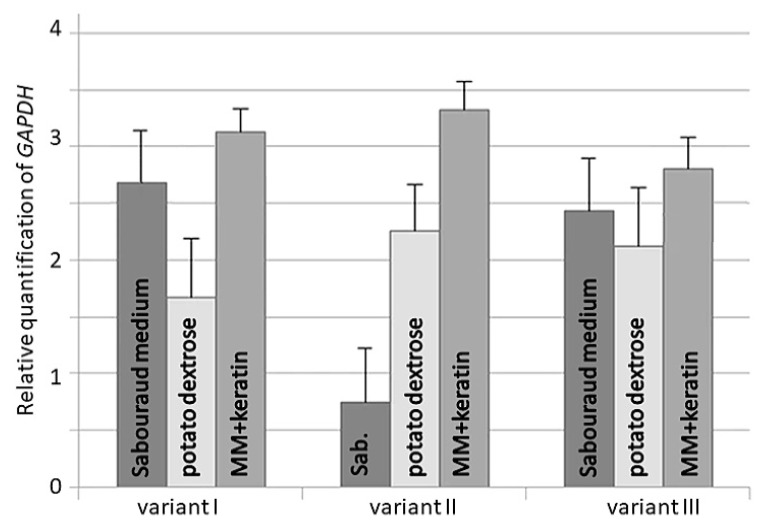
Relative quantification of *GAPDH* gene expression in *Trichophyton verrucosum* strains in Sabouraud glucose, potato dextrose, and MM-Cove supplemented with keratin media using different reference gene variants: I—two least stable reference genes *EEF1A1* and *ACTB*; II—three most stable reference genes *SDHA*, *TUBB*, and *RPL2*; III—all candidate reference genes. Error bars indicate standard deviation. Gene expression was studied using the 2^−ΔΔCt^ method.

The validation of the tested genes for the qRT-PCR method was analyzed in comparison to the expression profile of the *GAPDH* gene. The validation analysis was performed using three different reference gene variants selected by GeNorm, NormFinder, BestKeeper, and RefFinder in all *T. verrucosum* strains. The best result indicating an increase in *GAPDH* transcript was obtained when dermatophytes were grown in MM-Cove medium supplemented with keratin in relation to Sabouraud and potato dextrose media for the three tested variants. A comparable result indicating a simultaneous increase in *GAPDH* transcript and reference gens in three tested variants was obtained when dermatophytes were cultured in MM-Cove medium supplemented with keratin in relation to Sabouraud and potato dextrose media. The best result was revealed for *T. verrucosum* when grown in MM medium with keratin and genes tested in variant II (Figure 3).

## 4. Discussion

In the last several decades, an epidemiological renaissance of zoophilic dermatomycoses caused by a variety of factors has been noted. Therefore, the beginning of the era of molecular diagnostics in the field of medical mycology promised the quick and accurate identification of these fungi from clinical specimens [17,33]. Dermatologists and veterinarians agree that accurate species identification is the most important element of diagnostics, which prompts an appropriate choice of medical treatment [10,34].

An increasingly frequent method of species identification, also in mycology, is the quantification reverse transcription PCR technique (qRT-PCR) [23,26,35]. However, its need for adequate selection of reference genes is a limitation, which is associated with a strong dependence on the stability of the transcription level of these genes when in incubation conditions [26]. In many studies, normalized genes are regarded as those with stable expression in previously defined conditions; thus, they are appropriate to quantify the gene expression level of specific targets [15,26,36,37,38]. In agreement with our results, these studies reveal that no single gene is stably expressed in all microorganisms and in various growth conditions. Therefore, the expression stability of genes for identification purposes needs to be verified. In this study, we analyzed the expression stability of three genes (*TUBB*, *SDHA*, and *EEF1A1)* after the growth of *T. verrucosum* in Sabouraud and MM-Cove media. The former medium is used most commonly in mycological laboratories worldwide; the latter, used as minimal medium supplemented with bovine keratin, resembles the natural environment for keratinophilic dermatophytes. The gene expression stability was evaluated using geNorm, NormFinder, BestKeeper, and RefFinder algorithms.

Furthermore, these three genes selected for the analysis have already been studied, although very poorly, as identification targets for other fungal and dermatophytic species [26,39,40,41]. However, to our knowledge, this is the first identification and validation of the *TUBB*, *SDHA*, and *EEF1A1* genes as identification targets of *T. verrucosum* clinical isolates. The *SDHA* gene codes for a subunit of succinate dehydrogenase and is important in cellular respiration. In turn, the *EEF1A1* gene is responsible for encoding an isoform of the alpha subunit of the elongation factor-1 complex, which takes part in the enzymatic delivery of aminoacyl tRNAs to the ribosome. Finally, the *TUBB* gene is responsible for encoding one of the microtubule polymers, i.e., a major component of the eukaryotic cytoskeleton [42,43]. 

The succinate dehydrogenase complex flavoprotein subunit A (*SDHA*) gene was classified in our study as the most stably expressed gene, regardless of the dermatophyte growth conditions tested in this study. The lack of dependence of gene expression on the cultivation medium is important for at least two reasons, i.e., simplicity of the procedure and reliability of results. Currently, these features of identification techniques are recognized as the main benefits in the implementation of methods for routine use [15,17,33]. Similarly, Ciesielska et al. [26] reported that the *SDHA* gene was also a suitable reference gene for *M*. *canis* identification among a larger set consisting of nine candidates. Additionally, this gene was revealed as a suitable target for expression analyses in bovine tuberculosis [44], human glioma [45], horse cells under athletic stress [46], neutrophils, and untreated total blood leukocytes [47]. On the other hand, some reports showed that *SDHA* gene expression stability was low in yeast-like fungi, i.e., *Candida glabrata* [48] or different tissues of yak fetuses [49]. Therefore, although the *SDHA* gene is classified as a housekeeping gene, its transcription level may vary between different organisms, cell types or developmental stages, and growth conditions [50]. The other analyzed genes, *TUBB* and *EEF1A1*, showed statistically significantly lower expression stability assessed with the RefFinder algorithm for the *T. verrucosum* clinical isolates. Similar results were obtained for *M. canis*, which may suggest that the expression of these genes is so unstable that they are not suitable as identification targets in qRT-PCR for dermatophytes [26].

Interestingly, regardless of the host from which the clinical strain was isolated, the level of gene expression in cells incubated in minimal bovine keratin-supplemented medium and in Sabouraud medium remained at a similar level, with only slightly greater stability of gene expression in the former. Moreover, phenotypic analysis of the growth of clinical isolates of *T. verrucosum* in minimal medium with the addition of keratin of various animal species and human keratin showed significant differences in the growth of mycelium mass [28,51]. Probably, this variation is associated with the expression of metalloprotease genes responsible for keratinolytic activity and has no effect on the expression profile of *SDHA* and the other tested genes [52,53]. Finally, the reliability of the selected gene based on analysis of relative quantification of the *GAPDH* gene, which was induced at a relatively stable level, was validated. In this experiment, a similar expression level was demonstrated regardless of the growth conditions. Since *GAPDH*, together with *18S*, is most commonly used in qRT-PCR analysis as well as in the case of dermatophytes [39,54], it can be concluded that the *SDHA* gene can be an additional good candidate as an identification target in this technique. 

## 5. Conclusions

Our results have clearly proven that normalization of the expression stability of genes as potential candidates for identification targets in the qRT-PCR method can take into account the current standards of dermatophyte culture in the mycological laboratory. The incubation in Sabouraud medium makes it possible to perform multidirectional identification based on the assessment of dermatophyte morphology and direct use of the colony for molecular analysis without losing the reliability of the results obtained. These results may allow more profound analysis of *T. verrucosum* gene expression in various culture conditions routinely used in diagnostic laboratories while ensuring improved accuracy and reliability of new developed identification techniques.

## Figures and Tables

**Table 1:** Characterization of genes tested with qRT-PCR in clinical isolates of *Trichophyton verrucosum*.

Gene	Name	Primers (5′-3′)	Length of Product (bp)	Tm (°C)	Ct Range
*TUBB*	β-tubulin	AAGAGTTCCCAGACCGTT TGTTGTACAAGGCCTCAT	161	59.5	15.9–18.4
*ACTB*	β-actin	TCCTGAGGCTCTCTTCC GTAGTACCGCCGGACAT	144	60.0	16.3–18.8
*ADPRF*	ADP ribosylation factor	TTCTCATGGTCGGTCTC CGTTGAATCCGATGGTG	101	58.5	16.1–19.8
*RPL2*	ribosomal protein L2	GATCTATATTCACGGCTC ATGATGTTCTTCACGACA	111	60.5	16.4–19.3
*SDHA*	succinate dehydrogenase complex flavoprotein subunit A	TCTAGGAAACATGCACAT TTCGATAACACTCTGAGT	130	60.5	16.0–18.9
*EEF1A1*	elongation factor 1-α	CCTAAGTTCGTCAAGTCT CTTCTCGACAGCCTTGAT	161	60.0	16.2–19.1
*GAPDH*	glyceraldehyde-3-phosphate dehydrogenase	AACGGCTTCGGTCGTATTG TATTCGGCGTATTTGGTCTCA	110	59.5	15.8–18.7

**Table 2:** Expression stability of *Trichophyton verrucosum* genes determined by geNorm, NormFinder, BestKeeper, and RefFinder in the experimental conditions.

Gene	Condition	geNorm		NormFinder		BestKeeper			RefFinder	
		M-Value		SV-Value		r-Index		CV (%Ct)	Ranking Value	
*TUBB*	Sabouraud	0.593	0.561	0.256	0.231	0.832	0.78	8.18	6.732	6.329
	MM+keratin	0.489		0.203		0.721			6.021	
	potato dextrose	0.603		0.234		0.787			6.234	
*SDHA*	Sabouraud	0.497	0.455	0.098	0.092	0.886	0.887	8.57	2.120	2.083
	MM+keratin	0.413		0.087		0.854			2.013	
	potato dextrose	0.455		0.091		0.921			2.115	
*EEF1A1*	Sabouraud	0.765	0.746	0.403	0.36	0.907	0.906	8.47	7.234	7.112
	MM+keratin	0.712		0.299		0.890			6.983	
	potato dextrose	0.761		0.378		0.921			7.120	
*ACTB*	Sabouraud	0.595	0.581	0.377	0.349	0.968	0.934	8.54	6.321	6.128
	MM+keratin	0.546		0.304		0.879			5.954	
	potato dextrose	0.601		0.367		0.954			6.110	
*ADPRF*	Sabouraud	0.598	0.57	0.254	0.217	0.921	0.906	8.65	7.012	6.714
	MM+keratin	0.499		0.176		0.854			6.121	
	potato dextrose	0.612		0.221		0.943			7.010	
*RPL2*	Sabouraud	0.621	0.57	0.205	0.178	0.896	0.867	8.97	6.755	6.476
	MM+keratin	0.564		0.146		0.812			5.999	
	potato dextrose	0.605		0.185		0.901			6.675	

## Data Availability

All data are available from corresponding authors.
